# YinQiSanHuang Jiedu decoction for the treatment of hepatitis B-related compensated liver cirrhosis: study protocol for a multi-center randomized controlled trial

**DOI:** 10.1186/s13063-021-05650-6

**Published:** 2021-10-14

**Authors:** Qing-Juan Wu, Wen-Liang Lv, Juan-Mei Li, Ting-Ting Zhang, Wen-Hui Zhou, Qiang Zhang, Jiu-Chong Wang, Qing-Nan Wang, Zi-Ang Yao, Rui Qiang, Si-Tong Chen, Xin Zhao, Shuang Liu, Zheng-Min Cao, Lei Xu, Gao-Hui Li, Jing Chen, Li Wang

**Affiliations:** 1 China Academy of Traditional Chinese Medicine Guanganmen Hospital, Beijing, China; 2 Beijing University of Chinese Medicine, Beijing, China

## Abstract

**Introduction:**

Hepatitis B-related compensated liver cirrhosis is related to a higher risk of hepatocellular carcinoma, and antiviral therapy is the preferred method. As the pathological mechanisms of liver fibrosis are complex, drugs developed for a single target are difficult to be effective in clinical practice, so there are no chemical drugs or biological drugs with clear efficacy available for clinical application at present. Traditional Chinese medicine is a kind of medical science that has been gradually formed during thousands of years and continuously enriched by the people of all ethnic groups in China. Traditional Chinese medicine shows curative effects in the treatment of liver diseases, especially in the field of liver fibrosis prevention and treatment. This study aims to test the integrative medicine (Chinese medicine plus antiviral therapy) effective on lowing hepatocellular carcinoma risk among patients with hepatitis-related compensated liver cirrhosis.

**Methods and analysis:**

This is a multi-center randomized controlled trial, and a total of 5 hospitals and 802 patients will be involved in. All the subjects are randomly allocated to the YinQiSanHuang Jiedu decoction (YQSHD) group (*n* = 401) or the placebo group (*n* = 401). The YQSHD group receives YQSHD granule with entecavir (ETV), and the placebo group receives YQSHD placebo with ETV. The treatment period will last for 52 weeks, and the follow-up period for 52 ± 2 weeks. The primary outcome measure is the annual incidence of HCC. Outcomes will be assessed at baseline and after treatment. The objective of this trial is “the integrative of YQSHD with ETV reduce the annual incidence of HCC to 1%.”

**Ethics and dissemination:**

The protocol has been approved by the Medical Ethics Committee of Guang’anmen Hospital, China (No.2019-006-KY), and the other centers in the trial will not begin recruiting until the local ethical approval has been obtained. Trial final results will be disseminated via publication.

**Trial registration:**

Chinese Clinical Trial Registry ChiCTR1900021532. Registered on February 26, 2019

## Background

Hepatocellular carcinoma (HCC) is the second leading cause of cancer-related mortality worldwide, while liver cirrhosis (LC) is the main risk factor for HCC [1]. Liver fibrosis is involved in most chronic liver diseases, with further development could lead to liver cirrhosis, which affects the health and life of patients seriously [1]. A prospective study showed that the annual incidence of chronic hepatitis B (CHB) progressing to LC was 2~10% [2]. Therefore, active treatment of liver fibrosis, reversing or delaying its development to improve the prognosis of the disease and the quality of life of patients, has a very important significance. Nucleos(t)ide analogues (NAs) are the first-line treatment option for most patients with CHB, and the risk of developing HBV-related HCC is reduced by antiviral therapy [3]. Entecavir (ETV) is a kind of NAs recommended for HBV-related cirrhosis treatment, which is reported that it could relieve LC symptoms, improve patient prognosis, and prevent the development of HCC. It was reported that long-term treatment with ETV, lamivudine (LDV), or tenofovir (TDF) could reverse HBV-related cirrhosis to milder fibrosis [4]. However, the selection of resistant mutants and nephrotoxicity during long-term therapy limit its use [5, 6]. Therefore, the treatment goals for LC patients are to maximize long-term inhibition of HBV replication [7], reducing liver cell inflammation and necrosis and liver fibrous tissue hyperplasia, delaying and reducing the occurrence of liver failure, decompensation of LC, HCC, and other complications, improving the quality of life of patients, and extending their survival time, and for some patients could pursue the clinical cure [8, 9]. To achieve those treatment goals, many patients and internal medicine doctors seek supplemental and alternative therapies in addition to the antiviral methods, such as traditional Chinese medicine (TCM).

In China, about 85% of HCC occurring on the basis of LC, early prevention, early diagnosis, and early treatment are the keys to reducing incidence and mortality of HCC [10]. The purpose of treatment in the compensatory phase of LC is to control viral replication, inhibit disease progression, avoid complications, and reduce fibrosis. At present, there is no medicine that has been clinically and effectively verified for anti-liver fibrosis, but TCM has played an important role in delaying the disease progression [11]. A study demonstrated that long-term TCM use may attenuate LC risk in patients with CHB, the research results showed that TCM users had a significantly lower liver cirrhosis risk than TCM nonusers (adjusted HR = 0.416, *95% CI*, 0.231–0.749), and the histological evaluation revealed improved fibrosis in 45.0% of TCM users and 11.1% of TCM nonusers (=0.033). At the same time, the analyzation of the prescriptions including a total of 119 single Chinese herbs medicinal demonstrated that “replenish qi and fortify the spleen,” “clear heat and dispel dampness,” and “soothe the liver and regulate qi” are the main treatment methods of TCM for CHB [12]. Alisma Shugan Decoction (ASD), a kind of traditional Chinese medicine compound, ameliorates hepatotoxicity and associated liver dysfunction by inhibiting oxidative stress and p65/Nrf2/JunD signaling dysregulation in vivo [13]. Clinical study has reported that the integrative medicine therapy (ETV plus TCM) can promote the reduction of HBsAg level and the clearance of HBeAg in CHB patients with partial response to ETV through regulating the differentiation of B-cell subsets; another clinical research shows that combination therapy of traditional Chinese medicine plus ETV for 48 weeks resulted in a higher rate of necroinflammatory improvement and fibrosis regression than ETV alone in CHB patients with serious liver fibrosis/cirrhosis [14, 15]. The above researches have proven that TCM plays an important role in the prevention and treatment of liver fibrosis; however, there is still lacking of large-sample, multi-center randomized controlled studies to provide reliable and high-level evidence-based medical evidence for the prevention and treatment of liver fibrosis by TCM.

In TCM theory, the pathogenesis of LC is the deficiency of the essence; correspondingly, the main treatment principles include promoting blood circulation and removing blood stasis, strengthening the body tonic, clearing away heat, detoxifying, and removing dampness. *YinqiSanhuang Jiedu* decoction (YQSHD) is a traditional Chinese medicine compound, and the main components are *Huang Qi* (*Astragalus propinquus* Schischkin., Radix Astragali), *Yin Chen* (*Artemisia capillaris* Thunb., virgate wormwood herb capillary wormwood herb), and so on, as shown in Table 1. It shows the clinical effects of “clearing away heat,” “detoxifying and removing dampness,” “promoting blood circulation,” “removing stasis,” and “strengthening the body.” Which has been used clinically for many years, in 2018, we conducted a clinical observational study enrolled in 100 subjects. We observed two groups’ (combination group oral TCM plus ETV, and control group oral ETV only) CHB-related symptoms and signs, such as losing of appetite, fatigue, flank pain, yellowing of the body, fullness of the abdominal abdomen, dry eyes, nausea, belching, dull complexion, dry mouth, bitter mouth, loose stools, and frequent nocturia; results showed that the two groups were improving significantly with the extension of the treatment time. Besides, with the extension of the treatment period, the alanine aminotransferase (ALT), aspartate aminotransferase (AST), total bilirubin (TBIL), and gamma-glutamyl transferase (GGT) of the two groups decreased significantly, with the combination group dropping obviously than the control group. The normalization rate of ALT in the control group was 52%, and in the combination group, it was 94%; in terms of virological response, the HBV-DNA conversion rate in the control group was 62%, and the combination group was 86%; those differences between the two groups are statistically significant [16]. In the previous laboratory study, we had established the liver fibrosis model by intraperitoneal injection of 40% CCl_4_ (2 ml/kg) to Wistar rat for 4 weeks and extracted the three-dimensional imaging of live cells of primary rat liver sinusoidal endothelial cells (LSECs), which maximized the fidelity of the cell’s in vivo life state. Through the atomic force microscope (AFM) to “palp” the cells, introduce the concept of biopharmacology into the research. The results showed that the TCM group (intervened by astragalus polysaccharide (AP), which is the main active ingredient of the astragalus medicine in YQSHD) under the exposure mode of atomic force microscope LSECs spread more obviously, and Young’s modulus of LSECs after fibrotic serum injury was higher than the control group (the decrease in Young’s modulus means that the rigidity of the cells becomes smaller). At the same time, with the aid of a fast laser confocal fluorescence microscope/total internal reflection fluorescence microscope combined imaging system, the effect of AP on the secretion of NO in LSECs was observed. Medium and high concentrations of AP solutions can slow down the decrease in the amount of NO synthesized (NOs) in LSECs (those results have not yet been published). In order to test the effectiveness and safety of YQSHD and test its effect on delaying the progression of compensated liver fibrosis combined with ETV, we designed this multi-center, large-sample randomized controlled blinded trial. The purpose of the study is to reduce the annual incidence of compensated liver cirrhosis to HCC to 1%.

**Table 1:** Main components of *YinQiSanHuang Jiedu* decoction

Chinese name	Latin name	English name	Pharmacological action	Main active ingredient	The original producing area	Medicinal part	Amount/(%)
*Huang Qi*	*Astragalus propinquus* Schischkin	*Radix Astragali*	Promote liver cell growth, anti-liver fibrosis, antiviral, regulate immunity	Astragalus polysaccharide, Astragaloside (I, V, III), Calycosin	*Neimenggu*, China	Rhizome	10.81
*Yin Chen*	*Artemisia capillaris* Thunb.	Virgate wormwood herb capillary wormwood herb	Lower blood lipids to treat fatty liver, reduce alcoholic liver damage, inhibit the replication of hepatitis B virus DNA	Capillin, capillene, capillanol, capillarisin, 6,7-dimethylsculetin	*Shanxi*, China	Aboveground part of the plant	10.81
*Huang Qin*	*Scutellaria baicalensis* Georgi	Baical skullcap root	Anti-hepatocyte inflammation, anti-hepatocyte apoptosis, anti-hepatocyte mitochondrial lipid peroxidation, regulate immunity	Baicalein, neobaicalein, skullcapflavoneII, baicalin, wogonin	*Hebei*, China	Rhizome	2.7
*Huang Lian*	*Coptis chinensis* Franch.	Coptis root	Anti-hepatocyte mitochondrial lipid peroxidation, inhibit hepatoma cell proliferation, prevent liver fibrosis	Berberine, coptisine, epiberberine, berberrubine, palmatine	*Sichuan*, China	Tuber root	2.7
*Huang Bai*	*Platycladus orientalis* (Linn.) Franco	Bark of Chinese corktree	Inhibit immune response, selective inhibit HBAg, anti-inflammatory	Berberine, phellodendrine, magnoflorine, jatrorrhizine, palmatine	*Sichuan*, China	Dry bark	2.7
*E Zhu*	Curcuma aeruginosa Roxb.[C.zedoarianonRosc.]	Rhizome curcumae	Inhibit hepatoma cell proliferation, induced apoptosis of liver cancer cells, anti-liver fibrosis	Volatile oil (curzenone, borneo1, pormacrone), curcumene, cudione, turme	*Guangxi*, China	Tuber root	5.4
*Bie Jia*	*Trionyx sinensis* (Wiegmann)	Turtle shell	Anti-liver fibrosis, promote immunity, anti-hepatocyte injury	Collagen, trionyx sinesis polysaccharides, amino acid (aspartic acid, hreonine, glutamic acid), calcium carbonate, calcium phosphate	*Hubei*, China	Carapace	2.7
*Jiao Shan Zha*	*Crataegus pinnatifida* Bunge var.major N.E.Br.	Hawthorn fruit	Lower cholesterol, anti-bacterial, anti-hypertensive	Epicatechin, quercetin, hyperoside, chlorogenic acid, anthocyanin, ursolic acid	*Shandong*, China	Fruit	13.52
*Bai Shao*	*Paconia lactiflora* Pall.	Radix paeoniae alba	Anti-hepatocyte injury, anti-liver fibrosis, anti-fatty liver	Paeoniflorin, oxy-paeoniflorin, benzoylpaooniflorin, albi-florin, paeoniflorigenone	*Anhui*, China	Rhizome	10.81
*Ling Xiao Hua*	*Campsis grandiflora* (Thunb.) K.Schum.	Trumpet creeper flower	Anti-oxidation, inhibit thrombosis, anti-inflammatory	Apigenin, β-sitos-terol	*Jiangsu*, China	Flower	5.4
*Bai Zhu*	*Atractylodes macrocephala* Koidz.	Largehead Atractylodes Rhizome	Inhibit liver cancer cell metastasis, promote cellular immune function, inhibit the activating of metabolic enzymes	Volatile oil (humu-lene, β-elemol, α-curcumene, α-tractlone, 3β-acetoxyatractylone), Sesquiterpene lactone compounds (atractylenolide, 8β-ethoxyatractylenolide-II), Polyacetylene(14-acetyl-12-senecioyl-2E,8Z,10E-atracetylentriol)	*Zhejiang*, China	Tuber root	8.12
*Fu Ling*	*Poria cocos* (Schw.) Wolf.	Tuckahoe	Enhance cellular and humoral immunity, inhibit the DNA synthesis of tumor cell, inhibit hepatocyte necrosis, anti-tumor	Pachymic acid, tumulosic acid, pachymic acid methyl ester, pachy-man, Pachymaran	*Yunnan*, China	Dry sclerotia	8.12
*Chai Hu*	*Bupleurum chinense* DC.	Red Thorowax Root	Anti-liver fibrosis, inhibit acute liver injury, inhibit proliferation of liver cancer cells, promote apoptosis of liver cancer cells, anti-liver injury	Volatile oil (pentanoic acid, hexanoic acid, heptanoic acid, 2-heptenoic acid)	*Hebei*, China	Rhizome	5.4
*Bai Hua She She Cao*	*Hedyotis diffusa* Willd.	Spreading Hedyotis herb	Enhance hepatocyte immunogenic to anti-tumor, inhibit proliferation of liver cancer cells, promote apoptosis of liver cancer cells	Asperuloside, asperulosidic acid, deacetylasperulosidicacid, geniposidic acid, scandoside	*Guangxi*, China	Whole plant	10.81

## Methods

### Study setting and recruitment

A total of 802 patients will be recruited from 10 hospitals: Guang’anmen Hospital of China Academy of Chinese Medical Sciences is the responsible unit and will recruit 82 cases, XiXi Hospital of Hangzhou will recruit 80 cases, Shuguang Hospital Affiliated to Shanghai University of Traditional Chinese Medicine will recruit 80 cases, the Sixth People’s Hospital of Qingdao will recruit 80 cases, Nanjing Second Hospital will recruit 80 cases, Nanchang Ninth Hospital will recruit 80 cases, Beijing Shunyi Traditional Chinese Medicine Hospital will recruit 80 cases, the Sixth People’s Hospital of Shenyang will recruit 80 cases, Beijing Ditan Hospital Capital Medical University will recruit 80 cases, and Chengdu University of Chinese Medicine Affiliated Hospital will recruit 80 cases.

Outpatients in clinics are the main recruitment objects. Poster and online publicity with a brief introduction to the trial and the contact information of researchers will also be used for recruitment. Before enrollment, every participant will be provided with a complete and comprehensive description of the test procedure, purpose, potential adverse events, and expected benefits. All subjects will be evaluated during the screening period to test whether they meet the inclusion criteria, and they will be informed that they may withdraw from the trial anytime. The researchers will obtain informed consent or assent from potential trial participants or authorized surrogates. The screening evaluation includes the general situation, disease-related symptoms and signs, and corresponding laboratory tests, including urine pregnancy test (women of childbearing age), HBV-DNA, HBsAg, HBsAb, HBeAg, HBeAb, HBcAb, liver function, alpha-fetoprotein (AFP), liver B-ultrasound or MRI/CT, and other examinations.

### Eligibility criteria

#### Inclusion criteria

The inclusion criteria are as follows:

- Patients with hepatitis B-related compensatory liver cirrhosis
- Between 18 and 65 years old
- Patients show syndromes of liver stagnation and spleen deficiency and dampness in TCM. (For the TCM diagnostic criteria, we refer to “National Standards for TCM Clinical Diagnosis and Treatment of the People's Republic of China” [17] and the “Medical Consensus of diagnosis and treatment of cirrhosis with integrated TCM and Western medicine,” [18] which was published by Digestive System Diseases Committee, Society of Integrated Traditional Chinese and Western Medicine.)
- Voluntary signing of informed consent

#### Exclusion criteria

The exclusion criteria are as follows:

- Patients with liver cirrhosis caused by other chronic liver diseases
- Patients with acute and chronic hepatitis, autoimmune hepatitis, primary biliary cirrhosis, primary sclerosing cholangitis, genetic metabolic liver disease, drug or toxic hepatitis, and alcoholic liver disease with non-HBV hepatotropic virus infection
- Pregnant or lactating women or women planning to become pregnant during the study period
- Patients who are allergic to the test drugs
- Patients who have mental disorders that cannot cooperate with the study, or patients with epilepsy in unstable status
- Patients with severe systemic diseases related with the heart, brain, lung, kidney, and hematopoiesis
- Patients of alcoholism or with other unsuitable conditions that are not suitable for enrollment. For those patients who are already using TCM, we will not enroll them unless they have stopped using TCM for more than 3 months
- Other situations deemed unsuitable by the investigator

If the following conditions occur, the subjects should discontinue the trial: (a) poor compliance, irregularly taking medicine, failure to revisit or revisit on time; (b) some combined diseases or complications, or deterioration during the trial; (c) subject self-withdrawal; (d) combined other drugs, or not taking test drugs according to research regulations; (e) lost contact; and (f) cannot provide complete information. There are specific stopping criteria: (a) serious safety problems occurred during the test, and the test should be stopped in a timely manner; (b) the drug was found to have no clinical value during the trial, and the trial should be stopped to avoid delaying the effective treatment of subjects; (c) it is found in the trial that there is a major error in the clinical trial protocol and it is difficult to evaluate the effect of the drug; or a well-designed protocol with important deviations in the implementation, and it is difficult to continue to evaluate the efficacy and safety of the drug; (d) the funding supporter requested stopping (such as funding reasons, management reasons, etc.); (e) the State Food and Drug Administration of China ordered the trial to be stopped for some reason; (f) the test is suspended due to force irresistible reasons.

### Interventions

The test group (YQSHD group) receives YQSHD formula granules 5g (brewed with 150–200ml water before being taken) twice a day, combined with entecavir (H20100019, ChiaTai TianQing Pharmaceuticals in Jiangsu, China) 0.5mg once a day. The control group (placebo group) receives YQSH placebo formula granules 5g (brewed with 150–200ml water before being taken) twice a day, combined with entecavir 0.5mg once a day. The main compositions of YQSHD, totally 14 kinds of herb, are shown in Table 1. The test drugs are made into Chinese medicine formula granule. YQSHD placebo is made of excipients, thinners, coloring agents, flavoring agents, and fried malt, which are similar to YQSHD in shape, color, smell, and taste.

Other antiviral medicines or TCM with similar clinical efficacy must not be taken during the trial, such as TDF or LDV, if the un-antiviral medicines combined, record them in the “Case Report Form (CRF).” If the subjects need other treatments or concomitant care, they should contact the doctor in advance.

### Outcomes

#### Primary outcome

The primary outcome is the annual incidence of HCC (the examination items include alpha-fetoprotein (AFP), liver B-ultrasound test, or abdominal MRI/CT imaging).

The primary outcome is evaluated before the treatment, at the 52nd weeks of the treatment period, and at the 52nd ± 2 weeks of the follow-up period.

#### Secondary outcomes

The secondary outcomes include HBV-DNA-negative rate, HBsAg-negative rate, HBeAg seroconversion rate, liver function (ALT, AST, GGT, ALP, ALB, and TBIL), spleen thickness, and the evaluation scores of patients’ clinical symptoms.

**Fig. 1:**
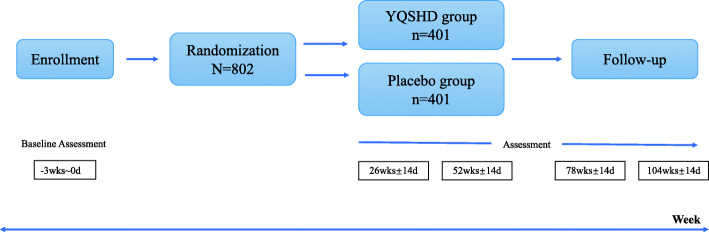
Flow diagram of the randomized, placebo-controlled, double-blinded trial of YQSHD for hepatitis B-related compensated liver cirrhosis

These indicators are observed before the treatment, at the 26th weeks and 52nd weeks of the treatment period, and at the 26th ± 2 weeks and 52nd ± 2 weeks of the follow-up period (Fig. 1).

#### Safety outcomes

The safety outcomes include the adverse events (AE), laboratory test (liver function, kidney function, blood routine test, urine routine test, et al.), electrocardiogram (ECG), basic vital signs, and physical examination.

**Fig. 2:**
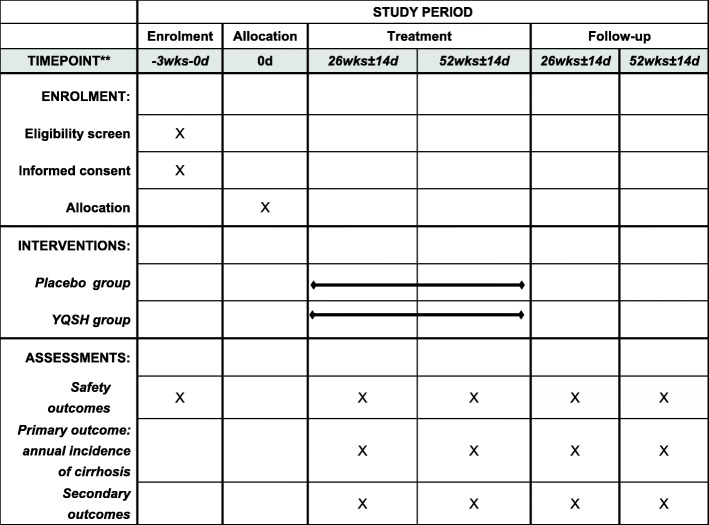
The schedule of enrollment, interventions, and assessments demonstrated in the Standard Protocol Items: Recommendations for Interventional Trials (SPIRIT) figure

And the basic vital signs are body temperature (T), blood pressure (BP), respiration (R), and heart rate (HR); laboratory tests include renal function tests, blood urea nitrogen (BUN), creatinine (Cr), blood, stool, and urine routine tests. These biological indicators are monitored from the baseline until the end of the follow-up (Fig. 2).

#### Participant timeline

The treatment period is 52 weeks and the follow-up period will last for 52 ± 2 weeks. We draw a flow diagram to make the timeline more clearly (Fig. 1).

#### Sample size

The aim of this study is to reduce the annual incidence of HCC from 3~6% [2] to 1% in CHB patients. Therefore, according to the sample size estimation formula for comparison of two sample rates, the incidence of target events is less than 0.2 (or 0.3) or greater than 0.8 (or 0.7), estimation formula as follows:

$$ n=\frac{{\left({u}_{\alpha }+{u}_{\beta}\right)}^2}{2{\left({\sin}^{-1}\sqrt{p_e-{\sin}^{-1}\;\sqrt{p_c}}\right)}^2} $$

The *p*_*e*_ and *p*_*c*_ represent the incidence rates of the test group (YQSHD group) and placebo group (control group), respectively; the positive event rate (*p*_*c*_) in the control group is 5%, while the target event rate (*p*_*e*_) in the test group is set to 1%. Since the values of and are small, so the degree is measured in radians, *α* = 0.05, *β* = 0.10. In this study, a two-sided test was chosen, *u*_0.05_ = 1.96, = 1.282, *p*_*e*_ = 0.05, and *p*_*c*_ = 0.01. The calculated sample size of each group is approximately 334 cases, allowing for 20% attrition; therefore, the total number of patients required for this trial is 334 × (1+20%) × 2 = 802 cases, with 401 in each group.

### Allocation

#### Sequence generation and implementation

In this study, the central randomization system (CRS) is used to centrally control the allocation of the entire randomization scheme. The randomized system mainly includes the following modules: subject screening, randomization, emergency blinding, drug formulation, drug supply management, and other functional modules. Central random principle: The researcher uses the screening module to enter some basic information of the subject (such as date of birth, gender) and obtain the subject’s unique identification number (SIN). Firstly, confirm patients with the inclusion criteria, log into the CRS, input the general information of the subjects, generate the random number, and fill in the electronic case report form (eCRF). Secondly, drug distributors apply for the drug number from CRS according to the random number. Finally, the drug senders verify the code on the drug package with the number in the system, then the drugs have been given to patients.

#### Concealment mechanism

The “central randomization” method was used to conceal the allocation: when researchers determined that the subjects meet criteria, the researchers log in to the central random system, enter some basic information of the subject, and obtain the subject’s SIN. Then, the central random system will assign subject random number and drug number according to the designed blind table. In order to make the blind method effective and reduce drug loss, the random numbers are separated from the drug numbers, but the corresponding treatment plans are consistent within the system.

#### Blinding and emergency unblinding

This is a double-blind trial. The blinding method is set up and implemented by the Medical Statistics Center of Tianjin University of TCM. Neither the study researchers nor the subjects know the medication grouping. In the course of the trial, there is a scientific and strict management implementation system and feasible operation methods. All the subjects are under a standardized observation with their clinical symptoms carefully recorded. Adverse reactions are carefully observed, and “emergency unblinding” is required for serious adverse reactions. A regular supervision, inspection, and return system ensures the implementation of the double-blinding method.

Unblinding would be at the end of the test to perform a statistical analysis of all the data. The outcome assessment will be blinded. When all the research data has been entered and locked, the third party participants who save the blinding codes and the researchers will jointly unblind and submit the database to the statistical analyst. When all the statistical analysis is completed, reports of statistical analysis and clinical trial summary are gonna be written by researchers.

#### Data collection and management

The investigator will prepare original documents for each subject who randomly entered the study, and information will be recorded in the CRF. All research results (including personal data, test documents, etc.) that appear in the original medical records will be completely confidential within the scope allowed by law. Not the full name but the name initials and the random number will be shown in CRF. The content should be comprehensive and accurate, so as to record all examination results and other relevant data. The research center shall keep these documents properly for 5 years after the end of the research. The researcher will authorize the relevant regulatory agency to directly access all research-related documents.

### Statistics analysis plan

#### Outcomes

For the statistical analysis of the comparison of the primary outcome incidence between the two groups, we will use the *χ*^2^ test and setting *P* <0.05 (95% confidence interval) as statistically significant; and for the secondary outcomes: where the measurement data is expressed as mean ± standard deviation, the count data is expressed as frequency and percentage (*f*, %), and the frequency or percentage of the efficacy evaluation index is converted into frequency and percentage (*f*, %). For the comparison of the mean between the two groups, the homogeneity test is performed first. If the variances are equal, the *t* test is used. If not discarded, the non-parametric *t*' test is used. The measurement data of each group before and after treatment is compared using the paired *t*/*t*' test. The comparison of grid table count data was performed using the *χ*^2^ test, and the comparison of rank data used the rank sum test. *P* < 0.05 was used as the statistical difference. The data analysis will be performed by SPSS 19.0 statistical software.

The baseline is defined as the last observation data before the first medication, which included demographic characteristics and clinical baseline data: age, sex, vital signs (height, weight, temperature, heart rate, blood pressure, breathing), clinical symptom score, HBV-DNA, AFP, entecavir treatment history, and CHB-related diseases.

#### Analysis population and missing data

##### Full analysis set (FAS)

According to the intentional therapy (ITT) principle, all randomized subjects’ data will enter the full analysis set. For subjects who withdraw from the study early for various reasons, the missing data will be filled by the way of last observation carry forward (LOCF).

##### Per-protocol set (PPS)

For those who enter the study and complete treatment and follow-up, the medication compliance is 80–120%, no combined medication that affected the effectiveness evaluation during the study period, with complete evaluation index data and no major test protocol violations, their indicator data will constitute the study’s per-protocol set.

##### Safety set (SS)

Safety set (SS) includes those subjects who received at least one treatment after randomization.

We have not plan to do subgroup analyses or sensitivity analyses currently.

There are principles for handling the follow-up losing: (a) if subjects’ loss is because of adverse reactions, the data will be recorded in the adverse reaction statistics; (b) if the loss is because of ineffectiveness, the data will be included in the efficacy statistics; (c) for those patients who were effective during the treatment but could not complete the entire course, and those lost to follow-up, these data will be included in the efficacy statistics and should be analyzed intentionally.

### Consent, harms, and AE

When the patient agrees to participate in the trial, the subject will sign two informed consent forms, kept by the patients and researchers, respectively. For those subjects who meet the criteria but with involuntary or incomplete autonomy, they can also enter the trial with the consent of the ethics committee, and the informed consent will be signed by their guardian.

Any adverse medical events that occur during treatment and follow-up, regardless of whether or not there is a causal relationship with the test medicines, should be considered as an adverse event (AE) and recorded in the CRF adverse event table specified. When filling out the AE report forms, it is necessary to detailed record the occurrence, time, severity, duration, measures taken, and outcomes of AE. If serious adverse events occur during the trial, emergency treatments should be taken immediately and reported to the responsible researcher of the trial, the ethics committees, and the China State Food and Drug Administration Safety Supervision Department within 24 h. All the adverse events should be tracked until the adverse symptoms disappear or the researchers confirm that further follow-up is no longer needed.

If the subject has an injury that is directly related to this study during the course of treatment, and it is confirmed by the medical identification, the research team will pay the subject medical expenses; for serious AE caused by drug-related injuries, the research team will give the injured subject certain compensation in accordance with relevant national laws and regulations, and the compensation costs will be borne by Guang’anmen Hospital.

### Monitoring and auditing

The composition of the data monitoring committee (DMC) will monitor the trial in accordance with the corresponding standard operating procedure, which is independent from the researchers. The DMC will be allowed to evaluate the quality and integrity of the study. Before this trial starts, uniform training should be conducted for all the researchers in clinical trials, including Good Clinical Practice (GCP), research protocols, Electronic Data Capture System (EDC), central stochastic systems, and the use of scales. The DMC will assess the capabilities of research centers and collect information about institutional facilities and technical equipment. During the period of the study, the DMC is responsible for verifying the clinical research records with the original records and resolving any problems that arise during the trial. The DMC will also monitor that the research centers adhere to the research protocol, arranges the supply of research drugs, and ensures that the drugs are kept under appropriate conditions in accordance with instructions. Each center should submit the main indicators to the clinical endpoint committee to be evaluated by the uniform standard. The principal investigator and authorized researcher should review, electronically sign, and date the eCRF. The DCM has access to interim results and makes the final decision with researchers to terminate the trial.

#### Adherence

At the trial beginning, the researcher would emphasize the importance of compliance to the subjects and require the subjects to bring back drug package (regardless of the remaining drugs) when they visited the research center. At the same time, we will establish an online platform to make immediate contact with the patient, and contact the patients at least twice a month to learn the patient’s situation and remind patients to actively return to the clinic. And for those patients who were effective during the treatment but could not complete the entire course, and those lost to follow-up, these data will be included in the efficacy statistics and should be analyzed intentionally.

Each research center will receive auditing visits every 3 months since the first patient is enrolled. The study will be regularly monitored by a Clinical Research Associate (CRA) in accordance with the corresponding standard operating procedure, they will help monitor whether written consent and dated informed consent forms (ICF) have been obtained from all subjects. A professional medical review would compare the data entered in the case report form (CRF) or eCRF with the original data, to ensure the quality of the data, the clinical logic, and general medical terms for the description. The researcher will properly keep the data to protect the rights and privacy of subjects, the documents in the clinical trial shall be preserved and managed in accordance with the requirements of the GCP, and the database will be maintained by EDC. The auditing procedures are independent from the investigators.

## Discussion and potential limitations

Studies have reported that the annual incidence of cirrhosis among CHB patients who have not received antiviral treatment is 2–10% [2], and a multi-center cohort study showed that the annual incidence of liver cancer in hepatitis B virus patients taking entecavir was 1.7% [3]. At the same time, studies reported that combined therapy is superior to conventional antiviral therapies [19–21], which not only can enhance the antiviral ability, on the other hand, it also can reduce the accompanying symptoms, improve the quality of life, and prolong the life of patients [22, 23]. Thus, the combination therapy could become a trendy of CHB treatment. To facilitate high validity and reliability, a strict quality control and high-quality methodology are indispensable. To facilitate appropriate high-quality methodology and strict quality control, this protocol has been developed according to the CONSORT statement [24] and SPIRIT 2013 [25]. This trial is based on the combination of the first-line antiviral drug ETV plus the TCM compound YQSHD. It is a multi-center, randomized, double-blind, placebo-controlled trial, and the purpose is to drop the annual liver cancer incidence among hepatitis B-related compensated liver cirrhosis patients to 1%. The results from this trial may provide evidence on the effectiveness and safety of YQSHD.

There are also some limitations to the study that should be considered. Due to restrictions in research project funds and trial period, the follow-up period could not be longer, and it is a pity that we cannot obtain follow-up data for 3 years, 5 years, or even longer. Besides, for the combination therapy, there are still some problems that should be clarified, such as what is the best time for combination therapy or whether it can be repeated after stopping the drug. Notwithstanding these limitations, the results from this study will provide new evidence about YQSHD from a well-designed trial. In addition, this study will provide a herbal prescription for adult CHB based on the guidelines for the diagnosis and treatment of liver fibrosis in integrative medicine practice (2019) [26].

### Trial status

The protocol version number is 1 and was finalized in October 2018. This protocol was registered in the Chinese Clinical Trial Registry (NO. ChiCTR1900021532; URL: http://www.chictr.org.cn/searchproj.aspx). The date of recruitment began on 23 October 2019. The original planning completed recruitment date is approximately December 2021; however, because of the sudden pandemic of COVID-19 in global, which will impact our enrollment seriously and induce to a delaying end date than we excepted. If it should amend the protocol, we will communicate with the investigators, ethics committee, trial registries, and other relevant parties.

### Ethics and dissemination

The protocol has been approved by the Medical Ethics Committee of Guang’anmen Hospital, China (which is the central ethical approval), and the other centers in the trial will not begin recruiting until the local ethical approval has been obtained. Trial final results will be disseminated via publication.

### Trial registration

ChiCTR1900021532, this protocol was registered in the Chinese Clinical Trial Registry (URL: http://www.chictr.org.cn/searchproj.aspx) on February 26, 2019.

## Data Availability

This is an Open Access article which permits others to distribute, remix, adapt, build upon this work non-commercially, and license their derivative works on different terms, provided the original work is properly cited and the use is non-commercial. Trial final results will be disseminated via publication.

## Abbreviations

AE: Adverse event
ALT: Alanine aminotransferase
AST: Aspartate aminotransferase
ALP: Alkaline phosphatase
ALB: Serum albumin
TBIL: Total bilirubin
BUN: Blood urea nitrogen
CHB: Chronic hepatitis B
CRS: Central Randomization System
CRF: Case Report Form
CHM: Chinese herbal medicine
DAAs: Antiviral agents
DMC: Data monitoring committee
ECG: Electrocardiogram
ETV: Entecavir
FDA: Food and Drug Administration
HBV: Hepatitis B virus
HCC: Hepatocellular carcinoma
LC: Liver cirrhosis
GCP: Good Clinical Practice
GGT: Gamma-glutamyl transferase
IFN: Interferon
NAs: Nucleos(t)ide Analogues
SIN: Subject’s unique identification number
TCM: Traditional Chinese medicine
YQSHD: YinQiSanHuang Jiedu decoction
